# Medication reminder use and medication adherence among patients with chronic conditions in a Malaysian teaching hospital

**DOI:** 10.1186/s12913-026-14457-7

**Published:** 2026-03-30

**Authors:** Nur Amalia Md Kasim, Umira Syahirah Shirinal Izuan, Mahmathi Karuppannan

**Affiliations:** 1 Department of Pharmacy Practice and Clinical Pharmacy, Faculty of Pharmacy, Universiti Teknologi MARA Cawangan Selangor, Kampus Puncak Alam, Bandar Puncak Alam, Selangor Darul Ehsan 42300 Malaysia; 2 Department of Pharmacy, Hospital Al-Sultan Abdullah, Universiti Teknologi MARA, Bandar Puncak Alam, Selangor 42300 Malaysia; 3 Telehealth and Remote Patient Care (TeleCare) Research Initiative Group, Universiti Teknologi MARA Cawangan Selangor, Kampus Puncak Alam, Bandar Puncak Alam, Selangor Darul Ehsan 42300 Malaysia

**Keywords:** Medication reminders, Medication adherence, Prevalence, Chronic diseases

## Abstract

**Background:**

Healthcare providers commonly recommend medication reminders as a strategy to enhance adherence to prescribed medications. Patient demographic characteristics may influence medication adherence and incorporating patient-centred approaches using reminders has the potential to improve health outcomes and quality of life.

**Objectives:**

This study aimed to (1) identify the types of medication reminders used by patients in their healthcare routines and (2) examine the association between medication reminder use and medication adherence.

**Methods:**

A cross-sectional study was conducted among patients aged 18 years and older who were prescribed chronic medications at a teaching hospital in Selangor, Malaysia. Data were collected using a validated questionnaire and the Malaysian Medication Adherence Assessment Tool (MyMAAT). Patients were recruited while waiting for medication refills or collection at the outpatient pharmacy department.

**Results:**

Of the 400 patients surveyed, only 28.5% (*n* = 114) reported using medication reminders, including alarms, mobile applications, written notes, or other methods. Medication reminder use was significantly associated with gender (*p* = 0.046) and highest education level (*p* = 0.006). Medication adherence was significantly associated with age, gender, education level, marital status, employment type, and medical history. However, no significant association was observed between medication reminder use and adherence, and the number of prescribed medications did not influence adherence levels.

**Conclusion:**

Fewer than one-third of patients reported using medication reminders, and no significant association was found between reminder use and medication adherence. Further research involving larger, multi-centered populations is needed to understand better the role of medication reminders in promoting adherence among patients with chronic conditions.

## Background

Medication adherence refers to “the extent to which the person’s behavior (including medication-taking) corresponds with agreed recommendations from a healthcare provider” [1]. Adherence is essential for ensuring the effectiveness, safety, and quality of medical interventions. Although often used interchangeably with compliance, adherence emphasizes a more active role of the patient, encompassing behavioral and lifestyle adjustments in addition to following prescribed regimens [2]. Conversely, compliance implies a more passive, instruction-following behavior.

Globally, average adherence to long-term therapy for chronic illnesses is estimated at approximately 50% in developed countries and is even lower in developing nations [1]. Medication non-adherence has been linked to poorer clinical outcomes and increased morbidity and mortality across various conditions, including human immunodeficiency virus (HIV) infection, cancer, diabetes, asthma, cardiovascular disease, and mental illness [3]. The economic burden of non-adherence is substantial, with estimated costs ranging from USD 949 to USD 44,190 per person annually, depending on the disease [4]. Lower adherence levels generally associated with higher overall healthcare expenditures, including increased costs related to inpatient care, outpatient services, pharmacy utilization, and emergency department visits.

In Malaysia, a scoping review reported that the prevalence of medication non-adherence among patients with type 2 diabetes ranged from 11.7% to 44.8% [5]. In contrast, a meta-analysis involving 37,429 older adults found a substantially higher non-adherence prevalence of 60.6% [6]. Additionally, a study conducted in an outpatient pharmacy department revealed that a total of 134 patients, 99% of whom were elderly (aged ≥ 60 years), returned unused medications valued at RM 13,594.90 (USD 3,354.70), corresponding to an average monthly cost of RM 1,132.90 (USD 279.56) [7]. Despite extensive subsidization of medication costs within the public healthcare system, medication non-adherence remains prevalent. Collectively, these findings indicate that adherence levels for chronic conditions in Malaysia are comparable to global estimates, highlighting the need for targeted interventions to address adherence challenges and reduce clinical and economic burdens.

Improving medication adherence is a key priority for healthcare providers, including physicians, nurses, as higher adherence is associated with better health outcomes, reduced healthcare costs, and decreased hospitalizations [8]. However, achieving optimal adherence remains challenging due to various factors, such as patients’ perceptions of medication efficacy, polypharmacy, complex treatment regimens, misunderstandings about medication instructions, forgetfulness, and concerns about potential side effects [9–12]. Consequently, non-adherence may lead to suboptimal clinical outcomes and increased strain on healthcare systems.

Numerous strategies have been employed to enhance medication adherence, including self-report measures, drug metabolite or biomarker testing, directly observed therapy (DOT), pill counts and pharmacy refill tracking and the use of digital tools such as medication reminders [13]. Medication reminders, whether in the form of alarms, mobile applications, written cues, or caregiver support, may assist patients in managing complex medication schedules. Nevertheless, there is no universally accepted approach to improving adherence, and the effectiveness of these interventions varies across populations and settings.

In Malaysia, several studies have evaluated medication reminder interventions, particularly mobile application-based approaches [14–16]. However, evidence describing the real-world prevalence of medication reminder use in routine practice, as well as the demographic and behavioral factors associated with reminder use and medication adherence among patients with chronic conditions, remains limited. Understanding these patterns is crucial for informing patient-centered adherence strategies. Therefore, this study investigates the types of medication reminders currently used by patients and examines the association between medication reminder use and medication adherence.

## Methodology

### Study design

This was a cross-sectional study conducted between March and May 2024 at the outpatient pharmacy department of a teaching hospital in Selangor, Malaysia. Data were collected using a validated survey instrument and the Malaysian Medication Adherence Assessment Tool (MyMAAT) [17]. The survey was researcher-assisted, with patients approached while waiting for medication refills or collection at the outpatient pharmacy. The study objectives and procedures were explained to all participants, and written informed consent was obtained prior to data collection. Ethical approval was granted by the Faculty of Pharmacy Research Ethics Committee, Universiti Teknologi MARA [REC (PH)/UG/112/2024] and Hospital Al-Sultan Abdullah, Universiti Teknologi MARA [500-HUiTM (PJI. 18/4/29)].

### Study population and sample size

The study population comprised patients aged 18 years and older who were prescribed at least one chronic medication. Participants were required to be able to read and understand Malay or English language. Patients receiving short-term medications, those unwilling to participate, and individuals with hearing, speech, or cognitive impairments were excluded from the study.

The required sample size was calculated using Yamane’s formula [18] with a 5% margin of error and a 95% confidence level, yielding in a minimum sample size of 399 participants. A convenience sampling approach was employed, and a total of 400 participants were recruited for the study.

### Study tool and data collection

A structured data collection form was used to gather demographic and clinical information, including medical registration number (MRN), medical history, and medication history. Participant responses were cross verified with their electronic medical records (EMRs) using their MRNs to ensure data accuracy. Information on medication reminders and medication adherence was collected using a structured questionnaire and the MyMAAT questionnaire [17]. The data collected comprised the following components:

1. **Medication reminder use**
  Participants were asked whether they used medication reminders and, if applicable, the type of reminder used (e.g., alarms, mobile applications, written notes, or caregiver support). The questions included:
  - *Are you using any reminders to take your prescribed medications?* (Yes/No)
  - *If yes to question (a)*,* what type of reminder(s) do you use?* (open-ended)
  - *If no*,* how likely are you to use any types of reminders in the future?* (Very likely, Somewhat likely, Somewhat unlikely, Very unlikely, Don’t know)

2) **Medication adherence**
  Medication adherence was assessed using MyMAAT [17], a validated 12-item instrument designed to measure adherence behaviors and related barriers. MyMAAT was developed and validated in Malaysia and is currently widely used in both clinical and research settings. The instrument is available in both English and Malay languages. Each item is scored on a five-point Likert scale, ranging from 5 (*strongly disagree*) to 1 (*strongly agree*), yielding a total score between 12 and 60. Patients were classified as adherent (scores 54–60) or non-adherent (scores 12–53). The tool demonstrated strong internal consistency with a Cronbach’s alpha of 0.91 [17].

### Data analysis

Data were analyzed using the Statistical Package for the Social Science (SPSS), version 29. Descriptive statistics, including frequencies, percentages, and means with standard deviations (SDs) were used to summarize demographic characteristics, medication reminder use, and medication adherence. Chi-square tests were performed to assess associations between medication reminder use and adherence status. Additionally, Spearman’s rank-order correlation analysis was conducted to examine the relationship between the number of prescribed medications and medication reminder use as well as medication adherence. Binary logistic regression followed by multivariate analysis was performed to identify factors independently associated with medication adherence. Results were reported as adjusted odds ratio (AORs) with 95% confidence intervals, and a statistical significance was set at *p* < 0.05.

## Results

A total of 400 patients participated in the study. The mean age of participants was 50 ± 18 years, with 38.8% (*n* = 155) aged 60 years and above. Most participants were female (65.0%, *n* = 260) and of Malay ethnicity (92.5%, *n* = 370). In terms of education attainment, 65.0% (*n* = 260) completed tertiary-level education, and 62.5% (*n* = 250) were married (Table 1). Employment status varied, with 34.0% (*n* = 136) being employed, 29.0% (*n* = 116) retired, and 16.0% (*n* = 64) students.

From clinical perspectives, most patients were receiving medications for chronic conditions, with asthma being the most frequently reported condition (12.5%, *n* = 50). Additionally, 60.7% (*n* = 243) of participants reported having multiple comorbidities. The majority of patients (84.8%, *n* = 339) were prescribed five medications, indicating a low prevalence of polypharmacy within the study population.

**Table 1:** Respondents’ demographic characteristics (*n* = 400)

Characteristics		*n* (%)
Age (years)	18–39	133 (33.3)
	40–59	112 (28.0)
	60 and above	155 (38.8)
Gender	Male	140 (35.0)
	Female	260 (65.0)
Race	Malay	370 (92.5)
	Chinese	12 (3.0)
	Indian	15 (3.8)
	Other^a^	3 (0.8)
Highest education level	No formal education	9 (2.3)
	Primary	16 (4.0)
	Secondary	115 (28.7)
	Tertiary	260 (65.0)
Marital status	Married	250 (62.5)
	Single	120 (30.0)
	Divorced	5 (1.3)
	Widowed	25 (6.3)
Type of employment	Employed	136 (34.0)
	Unemployed	36 (9.0)
	Retired	116 (29.0)
	Student	64 (16.0)
	Housewife/househusband	48 (12.0)
Medical history^b^	Asthma	50 (12.5)
	Diabetes	34 (8.5)
	Depression	24 (6.0)
	Hypertension	22 (5.5)
	Cancer	14 (3.5)
	High cholesterol	10 (2.5)
	Arthritis	8 (2.0)
	Heart disease	7 (1.8)
	Hyperthyroidism	7 (1.8)
	Gastritis	7 (1.8)
	Bipolar disorder	6 (1.5)
	Sinusitis	6 (1.5)
	Other^c^	39 (9.8)
Comorbidities (Mean = 1.92, SD = 0.948)	One	157 (39.3)
	More than one	243 (60.7)
Polypharmacy (Mean = 2.60, SD = 2.194)	No (< 5 total medications)	339 (84.8)
	Yes (≥ 5 total medications)	61 (15.3)

^a^includes Iban dan Kadazan from East Malaysia

^b^one patient could have more than one medical conditions

^c^psoriasis (5), stroke (4), chronic kidney disease (4), eczema (4), Parkinson (4), hypothyroidism (3), glaucoma (3), anemia (2), anxiety (2), nephrotic syndrome (2), prostate syndrome (2), systemic lupus erythematosus (2), ulcerative colitis (2)

### Prevalence of medication reminder use and medication adherence

Only 28.5% (*n* = 114) of patients reported using medication reminders (Table 2). Among these participants, the most commonly used reminders were phone alarms (32.2%, *n* = 38), caregiver assistance (23.7%, *n* = 28), pillboxes (13.6%, *n* = 16), physical calendars (12.7%, *n* = 15), and notes on whiteboards or diaries (11.0%, *n* = 13). In contrast, fewer participants reported using digital calendars (2.5%, *n* = 3) or health applications (2.5%, *n* = 3). Notably, 54.8% (*n* = 219) of participants were classified as non-adherent based on their MyMAAT scores, with an overall mean score of 49.72 ± 9.61.

**Table 2:** Respondents’ medication reminder use and medication adherence status

Variables		*n* (%)
Medication reminder use	Yes	114 (28.5)
	No	286 (71.5)
Type of medication reminder (*n* = 114)*	Phone alarm	38 (32.2)
	Caregiver/family members	28 (23.73)
	Pillbox	16 (13.56)
	Physical calendar	15 (12.71)
	Notes in diary/whiteboard	13 (11.02)
	Digital calendar	3 (2.54)
	Health app	3 (2.54)
	Refer prescription	2 (1.69)
Medication adherence (Mean = 49.72, SD = 9.61)	Adherent (score: 54–60)	181 (45.3)
	Not adherent (score: 12–53)	219 (54.8)

*Some patients may have used more than one medication reminders, so the total will not add up to 114

Further analysis identified factors associated with medication non-adherence (Table 3). No significant differences in non-adherence were observed between genders, with mean MyMAAT scores of 52.49 for males and 48.23 for females. Across age groups, older adults aged 60 years and above demonstrated the highest level of medication adherence, with a mean MyMAAT score of 54.06, which was higher than that observed in younger age groups.

Medication reminder usage was further examined across demographic and clinical variables. Most participants who reported using medication reminders were aged 60 years and above (*n* = 49, 43.0%), female (*n* = 65, 57.0%), and predominantly of Malay ethnicity (*n* = 109, 95.6%). The majority had attained tertiary-level education (*n* = 70, 61.4%), were married (*n* = 77, 67.5%), and were employed (*n* = 36, 31.6%). With respect to medical and medication history, most reminder users were being treated for asthma (*n* = 10, 8.8%), reporting having multiple comorbidities (*n* = 26, 55.3%), and were prescribed fewer than five medications (*n* = 95, 80.5%).

**Table 3:** Characteristics of respondents using medication reminder and adherence scores

Characteristics		MyMAAT Score Mean (SD)	Medication reminder use (*n*, %)
Age (years)	18–39	44.51 (9.049)	31 (27.2)
	40–59	49.91 (10.471)	34 (29.8)
	60 and above	54.06 (6.910)	49 (43.0)
Gender	Male	52.49 (8.384)	49 (43.0)
	Female	48.23 (9.910)	65 (57.0)
Race	Malay	49.52 (9.602)	109 (95.6)
	Chinese	54.83 (6.422)	2 (1.8)
	Indian	54.00 (7.874)	2 (1.8)
	Other	32.25 (2.121)	1 (0.9)
Highest education level	No formal education	51.67 (7.730)	2 (1.8)
	Primary	54.06 (5.627)	11 (9.6)
	Secondary	51.09 (9.604)	31 (27.2)
	Tertiary	48.78 (9.749)	70 (61.4)
Marital status	Married	52.03 (9.048)	77 (67.5)
	Single	44.46 (9.010)	29 (25.4)
	Divorced	53.20 (9.550)	2 (1.8)
	Widowed	51.20 (8.362)	6 (5.3)
Type of employment	Employed	48.93 (9.628)	36 (31.6)
	Unemployed	49.53 (10.363)	14 (12.3)
	Retired	53.23 (8.052)	34 (29.8)
	Student	42.55 (8.173)	15 (13.2)
	Housewife/househusband	53.21 (8.661)	15 (13.2)
Medical history	Asthma	44.00 (8.031)	10 (8.8)
	Diabetes	51.41 (10.638)	7 (6.1)
	Depression	52.91 (6.746)	5 (4.4)
	Hypertension	50.00 (11.343)	8 (7.0)
	Cancer	44.79 (9.885)	4 (3.5)
	High cholesterol	48.75 (15.327)	2 (1.8)
	Arthritis	55.29 (6.075)	2 (1.8)
	Heart disease	45.87 (9.589)	5 (4.4)
	Hyperthyroidism	44.00 (12.410)	1 (0.9)
	Gastritis	47.00 (15.567)	1 (0.9)
	Bipolar disorder	36.33 (23.544)	3 (2.6)
	Sinusitis	50.00 (6.525)	2 (1.8)
	Other	49.21 (8.154)	17 (9.6)
Comorbidities	One only	52.51 (8.656)	21 (44.7)
	More than one	52.22 (7.695)	26 (55.3)
Polypharmacy	No (< 5 total medications)	49.23 (9.870)	95 (80.5)
	Yes (≥ 5 total medications)	52.44 (7.527)	23 (19.5)

### Association between medication reminder use and medication adherence

Among the 114 patients who reported using medication reminders, 48.2% (*n* = 55) were classified as adherent, compared to 44.1% (*n* = 126) among non-users (Table 4). Chi-square analysis revealed no significant association between medication reminder use and adherence status [χ² (1, *n* = 400) = 0.578, *p* = 0.447].

**Table 4:** Correlation between medication reminder use and medication adherence

Statements	Adherent (*n* = 181)	Not adherent (*n* = 219)	χ2
Patients using medication reminder	55	59	0.578 *p* = 0.447
Patients not using medication reminder	126	156	

χ2 – Chi-square

### Correlation between number of medications, reminder use, and adherence

Spearman’s rank-order correlation analysis (Table 5) revealed a weak positive correlation between the number of medications and both medication reminder use (rs = 0.217, *p* = 0.021) and medication adherence (rs = 0.181, *p* < 0.001). These findings indicate that patients prescribed a greater number of medications were slightly more likely to use medication reminders and demonstrate higher adherence to their prescribed regimens.

**Table 5:** Correlation between number of medications with medication adherence and medication reminder use

Variables		Spearman rho (rs)	*p*-value
Number of medications	Medication reminder use	0.217	0.021
	Medication adherence	0.181	< 0.001

### Factors associated with medication adherence

Logistic regression analysis identified age, gender, and employment status as significant predictors of medication adherence (Table 6). The model correctly classified 71.5% of adherence and non-adherence cases. In contract, medication reminder use and the number of prescribed medications were not statistically significant predictors of medication adherence. Collectively, the model explained 29.3% of the variance in medication adherence (Nagelkerke 𝑅² = 0.293).

Age emerged as a significant predictor of medication adherence (OR = 2.720, 95% CI: 1.628–4.545; *p* < 0.001), indicating that older patients had significantly higher odds of medication adherence. In particularly, higher adherence was observed among patients aged 60 years and above. Gender was also significantly associated with medication adherence, with female patients demonstrating higher adherence compared with males (OR = 1.827, 95% CI: 1.091–3.058; *p* = 0.022), corresponding to 1.83 times greater odds of adherence.

Employment status was also associated with medication adherence, although the effects varied across categories. Students had significantly lower odds of adherence compared to employed individuals (OR = 0.300, *p* = 0.025). In contrast, other employment categories, including retired individuals, housewives/househusbands, and the unemployed, were not significantly associated with medication adherence (*p* > 0.05). Similarly, medication reminder use and the number of prescribed medications were not significant predictors of adherence (*p* > 0.05).

**Table 6:** Factors associated with medication adherence

Variables		Coef (B)	S.E.	Odds Ratio	95% CI		*p*-value
					Lower	Upper	
Age		1.001	0.262	2.720	1.628	4.545	< 0.001
Gender	Male			-			1.000
	Female	0.603	0.263	1.827	1.091	3.058	0.022
Type of employment	Employed			-			0.077
	Retired	-0.312	0.394	0.732	0.338	1.583	0.428
	Housewife/ househusband	0.064	0.469	1.066	0.425	2.672	0.891
	Student	-1.205	0.536	0.300	0.105	0.856	0.025
	Unemployed	-0.839	0.495	0.432	0.164	1.139	0.090
Medication reminder use	No						1.000
	Yes	-0.103	0.264	0.902	0.538	1.513	0.695
Number of medications		0.018	0.055	1.018	0.915	1.133	0.740

In the multivariate logistic regression analysis (Table 7), both age and gender were independently associated with medication adherence. Increasing age was associated with higher odds of adherence (adjusted OR = 1.05, 95% CI: 1.04–1.07, *p* < 0.001). Female patients were more likely to be adherent than male patients (adjusted OR = 1.71, 95% CI: 1.08–2.71, *p* = 0.022).

**Table 7:** Multivariate logistic regression analysis of factors associated with medication adherence

Variables		Coef (B)	S.E.	Odds ratio	95% CI		*p*-value
					Lower	Upper	
Age		0.050	0.007	1.052	1.038	1.066	< 0.001
Gender	Male			-			1.000
	Female	0.537	0.235	1.711	1.080	2.710	0.022

## Discussion

This study examined the prevalence and types of medication reminders used by patients with chronic conditions and their association with medication adherence. Additionally, factors associated with medication adherence among patients attending outpatient pharmacy department of a teaching hospital were explored. The findings revealed that less than one-third (28.5%) of patients reported using medication reminders, while more than half (54.8%) were classified as non-adherent based on MyMAAT scores. These results highlight the persistent challenges of achieving optimal medication adherence in the management of chronic conditions.

These findings are comparable to a study conducted in Singapore, which reported very low uptake of medication reminder applications, with only 2.6% of adults aged 62 years and above using or intending to use such tools [8]. Although the prevalence of medication reminder use was higher in the present study (28.5%), both findings indicate suboptimal adoption of medication reminders among patients with chronic conditions. Despite this difference in uptake, medication adherence remained low in the present study, with only approximately 45% of respondents adhering to their medications, regardless of medication reminder use. Similar adherence challenges have been reported in other populations, including patients with hematological-oncological chronic conditions, where non-adherence prevalence of approximately 50% were documented [19].

Among the patient who reported using medication reminders, phone alarms were the most commonly used modality, accounting for 32.2% of reminder users. Despite this, no significant association was observed between the type of medication reminder used and medication adherence. This finding suggests that the use of phone alarms or other reminder modalities does not necessarily translate into improved adherence outcomes. Furthermore, the study demonstrated no overall significant association between medication reminder use and medication adherence. One possible explanation is that some patients may maintain adequate adherence by integrating medication-taking into established daily routines [20], thereby reducing reliance on external reminder tools. These findings indicate that while medication reminders may support medication-taking behavior for some individuals, they are unlikely to be effective in isolation without addressing other behavioral and contextual factors influencing adherence [21].

Further evidence supports the potential role of medication reminders in improving adherence in specific contexts. A randomized clinical trial involving college students prescribed antidepressants demonstrated improved medication adherence with the use of a reminder application [22]. Similarly, a study conducted in South Korea reported that smart pill bottles significantly enhanced adherence among women with breast cancer [23]. However, the effectiveness of medication reminders may not be sustained over time. For example, a randomized controlled trial in Singapore found that short messaging service (SMS) reminders were effective in improving adherence only in the short term [24]. Taken together with evidence from randomized controlled trials and systematic reviews [25], these findings suggest that while mobile app–based reminder interventions can improve medication adherence in certain chronic conditions, their effectiveness is highly dependent on intervention features, duration of use, and patient characteristics. This may explain the absence of a significant association between medication reminder use and adherence observed in routine clinical practice in the present study.

Patients prescribed with a higher number of medications, indicative of polypharmacy, were more likely to use medication reminders and demonstrate higher levels of adherence in this study. This finding is consistent with a multicenter cross-sectional study conducted among older adults with multiple comorbidities and polypharmacy, which reported similar patterns of adherence behavior [26]. However, the relationship between medication burden and adherence is complex. A study examining the Medication Regimen Complexity Index (MRCI), which incorporates factors beyond the number of medication, found that patients with high regimen complexity (high MRCI scores) exhibited lower adherence compared with those with low or moderate complexity [27]. These findings highlight the importance of optimizing medication regimens to support adherence while minimizing the potential challenges associated with polypharmacy.

Interestingly, this study found that age was significantly associated with medication adherence, with higher adherence observed among older patients compared with younger individuals. This pattern is consistent with findings from previous studies, which have reported lower adherence rates among younger populations [19, 28]. Older adults may demonstrate better adherence due to more established daily routines [20], greater health awareness, and a higher likelihood of managing chronic conditions that require long-term treatment.

Gender also emerged as an important factor associated with medication adherence, with female participants demonstrating higher adherence rates than their male counterparts. This finding is consistent with a study among patients with arterial hypertension, which reported lower adherence with antihypertensive therapy among male patients [29]. Similarly, research conducted at Zagazig University Hospital found that female patients exhibited higher adherence rates compared to male patients [30]. However, the literature presents with mixed evidence, as a retrospective observational study involving 25,776 patients with chronic heart failure reported no significant gender differences in medication adherence, while other studies observed higher adherence rates among male patients [31].

The inconsistency in gender-related findings on medication adherence may be attributed to a range of physical, mental, and environmental factors [32]. For instance, a prospective multicenter study reported that women are more likely to experience medication-related side effects due to their complex physiological and hormonal changes, including those associated with menopause, pregnancy, and menstruation [33]. Adverse effects such as rashes, nausea, and diarrhea may disrupt daily functioning, and contribute to medication non-adherence. These physiological differences, together with lifestyle factors and adherence- assessed by tools such as MyMAAT, highlight the complexity of gender as a determinant of medication adherence.

Additionally, gender-specific illness perceptions may play a significant role in adherence behavior. Men are often guided by internal physiological cues and may be more likely to attribute ongoing symptoms to ineffective medications, potentially leading reduced adherence when perceived benefits are not immediate [34]. In contrast, women may rely more on external cues, such as blood pressure readings or healthcare provider recommendations, and may attribute illness to imbalances in lifestyle factors. However, women’s greater susceptibility to medication side effects may also influence their perception of personal control and subsequent adherence behaviors [32]. Collectively, these physiological, social, and psychological differences, alongside environmental influences, and treatment complexity, highlight the multifaceted role of gender in medication adherence.

Employment type may also influence medication adherence, although the association was not statistically significant in this study. The findings suggest that students, particularly those in the university settings, were more likely to be non-adherent compared with other employment groups. This may be attributed to busy lifestyles, irregular schedules, and competing academic and social demands, which can make consistent medication-taking challenging [35, 36]. Supporting this evidence, a study conducted among medical students at a private medical college in Bangladesh reported a high prevalence of self-medication practices [37]. Among the 204 students surveyed, only 28.4% reported adhering to physicians’ advice to continue prescribed medication when symptoms persisted, indicating a low adherence within this population. Tailored interventions that address time constraints and self-medication behaviors may be particularly important for improving adherence among students.

This study has several limitations that should be acknowledged. First, the demographic profile of the respondents was uneven, with a higher proportion of female participants and a predominance of Malay ethnicity, while representation from Chinese, Indian, and other ethnic groups was limited. As a result, the findings may not be generalizable to the broader Malaysian population. Additionally, the use of a convenience sampling method may have introduced selection bias, as participants were recruited based on accessibility and willingness to participate, potentially limiting representativeness of the sample. Furthermore, medication adherence was assessed using self-reported measures, which may not accurately reflect actual adherence behaviors. Response bias may also have influenced the findings, as some participants may have been reluctant to disclose non-adherent behaviors. Future studies should include larger and more diverse multi-ethnic populations and incorporate objective measures of adherence, such as pharmacy refill data, pill counts or electronic monitoring to enhance the robustness and generalizability of the findings.

The findings of this study have important implications for clinical practice, health systems, and future research. The association of age and gender with medication adherence highlights the need for targeted, patient-centered adherence strategies, particularly for younger and male patients. Routine use of validated tools such as MyMAAT may facilitate early identification of non-adherence in clinical settings. Additionally, understanding patients’ real-world use of medication reminders can inform the development of practical and acceptable adherence interventions. At the health system level, improving adherence has the potential to reduce medication wastage and healthcare costs. Future research should explore longitudinal and interventional approaches to evaluate tailored reminder strategies across different patient subgroups.

## Conclusion

This study demonstrates that the use of medication reminder among patients with chronic conditions remains low (28.5%), alongside a high prevalence of medication non-adherence (54.8%). Age and gender were significantly associated with medication adherence, whereas no significant association was observed between medication reminder use and adherence. Phone alarms were the most commonly used medication reminder (32.2%). While reminders alone may not improve adherence, they remain a valuable tool when integrated with comprehensive strategies addressing individual barriers. Targeted interventions, particularly for younger patients and those with demanding schedules, could enhance adherence and improve health outcomes. Further research, including multicenter and longitudinal studies, is warranted to better understand and address adherence challenges in diverse patient populations.

## Data Availability

All data generated or analysed during this study are included in this published article.

## References

[1] Sabaté E. Adherence to long-term therapies: evidence for action. Geneva: Switzerland: World Health Organization (WHO); 2003.

[2] Mir TH. Adherence Versus Compliance. HCA Healthc J Med. 2023;4(2):219–20. 37424969 10.36518/2689-0216.1513 PMC10324868

[3] Stewart SF, Moon Z, Horne R. Medication nonadherence: health impact, prevalence, correlates and interventions. Psychol Health. 2023;38(6):726–65. 36448201 10.1080/08870446.2022.2144923

[4] Cutler RL, Fernandez-Llimos F, Frommer M, Benrimoj C, Garcia-Cardenas V. Economic impact of medication non-adherence by disease groups: a systematic review. BMJ Open. 2018;8(1):e016982. 29358417 10.1136/bmjopen-2017-016982 PMC5780689

[5] Teng CL, Chan CW, Wong PS. Medication adherence of persons with type 2 diabetes in malaysia: a scoping review and meta-analysis. J ASEAN Fed Endocr Soc. 2022;37(1):75–82. 35800597 10.15605/jafes.037.01.14 PMC9242658

[6] Chang CT, Ang JY, Islam MA, Chan HK, Cheah WK, Gan SH. Prevalence of drug-related problems and complementary and alternative medicine use in Malaysia: a systematic review and meta-analysis of 37,249 older adults. Pharmaceuticals (Basel). 2021;14(3). 10.3390/ph14030187 PMC7996557 33669084

[7] Jamaluddin NA, Kamil TKTM, Ying MHW, Elnaem MH, Ahmed AM, Nazar NIM, et al. Types and costs of medications returned by outpatients at a Malaysian teaching hospital: a one-year cross-sectional study. J Pharm. 2022;2(2):141–8.

[8] Ping Y, Visaria A, Suppiah SD, Tan YW, Malhotra R. Prevalence and correlates of medication reminder app ‘use and use intention’ among older adults. Explor Res Clin Soc Pharm. 2022;6:100150. 35755719 10.1016/j.rcsop.2022.100150 PMC9218158

[9] Atinga RA, Yarney L, Gavu NM. Factors influencing long-term medication non-adherence among diabetes and hypertensive patients in Ghana: A qualitative investigation. PLoS ONE. 2018;13(3):e0193995. 29590156 10.1371/journal.pone.0193995 PMC5874015

[10] Planel-Ratna C, Juwaheer TD. Assessing the impact of digitalization and technology on patient compliance in healthcare services. High Tech Innov J. 2021.

[11] Kavitha S, Nalini GK, Suresh RM, Sahana GN, Deepak P, Nagaral JV. Treatment adherence and factors contributing to non adherence among type 2 diabetes mellitus patients in a tertiary care hospital: a cross sectional study. Int J basic Clin Pharmacol. 2017;6:689–94.

[12] Ge L, Heng BH, Yap CW. Understanding reasons and determinants of medication non-adherence in community-dwelling adults: a cross-sectional study comparing young and older age groups. BMC Health Serv Res. 2023;23(1):905. 37620970 10.1186/s12913-023-09904-8 PMC10464472

[13] Mason M, Cho Y, Rayo J, Gong Y, Harris M, Jiang Y. Technologies for Medication Adherence Monitoring and Technology Assessment Criteria: Narrative Review. JMIR Mhealth Uhealth. 2022;10(3):e35157. 35266873 10.2196/35157 PMC8949687

[14] Abdulrahman SA, Rampal L, Ibrahim F, Radhakrishnan AP, Kadir Shahar H, Othman N. Mobile phone reminders and peer counseling improve adherence and treatment outcomes of patients on ART in Malaysia: A randomized clinical trial. PLoS ONE. 2017;12(5):e0177698. 28520768 10.1371/journal.pone.0177698 PMC5433794

[15] Chew S, Lai PSM, Ng CJ. Usability and Utility of a Mobile App to Improve Medication Adherence Among Ambulatory Care Patients in Malaysia: Qualitative Study. JMIR Mhealth Uhealth. 2020;8(1):e15146. 32003748 10.2196/15146 PMC7055750

[16] Low PT, Ng CG, Kadir MS, Tang SL. Reminder through mobile messaging application improves outpatient attendance and medication adherence among patients with depression: An open-label randomised controlled trial. Med J Malaysia. 2021;76(5):617–23. 34508365

[17] Hatah E, Rahim N, Makmor-Bakry M, Mohamed Shah N, Mohamad N, Ahmad M, et al. Development and validation of Malaysia Medication Adherence Assessment Tool (MyMAAT) for diabetic patients. PLoS ONE. 2020;15(11):e0241909. 33157549 10.1371/journal.pone.0241909 PMC7647074

[18] Yamane T, Statistics. An Introductory Analysis. 2nd ed. New York: Harper and Row; 1967.

[19] Bouwman L, Eeltink CM, Visser O, Janssen J, Maaskant JM. Prevalence and associated factors of medication non-adherence in hematological-oncological patients in their home situation. BMC Cancer. 2017;17(1):739. 29121889 10.1186/s12885-017-3735-1 PMC5679497

[20] Klinedinst TC, Opsasnick L, Benavente JY, Wolf M, O’Conor R. The Roles of Busyness and Daily Routine in Medication Management Behaviors Among Older Adults. J Appl Gerontol. 2022;41(12):2566–73. 35950560 10.1177/07334648221120246 PMC9671821

[21] Fenerty SD, West C, Davis SA, Kaplan SG, Feldman SR. The effect of reminder systems on patients’ adherence to treatment. Patient Prefer Adherence. 2012;6:127–35. 22379363 10.2147/PPA.S26314 PMC3287416

[22] Hammonds T, Rickert K, Goldstein C, Gathright E, Gilmore S, Derflinger B, et al. Adherence to antidepressant medications: a randomized controlled trial of medication reminding in college students. J Am Coll Health. 2015;63(3):204–8. 25338175 10.1080/07448481.2014.975716

[23] Park HR, Kang HS, Kim SH, Singh-Carlson S. Effect of a Smart Pill Bottle Reminder Intervention on Medication Adherence, Self-efficacy, and Depression in Breast Cancer Survivors. Cancer Nurs. 2022;45(6):E874–82. 34661562 10.1097/NCC.0000000000001030 PMC9584037

[24] He Y, Tan EH, Wong ALA, Tan CC, Wong P, Lee SC et al. Improving medication adherence with adjuvant aromatase inhibitor in women with breast cancer: study protocol of a randomised controlled trial to evaluate the effect of short message service (SMS) reminder. BMC Cancer [Internet]. 2018;18(1):727. Available from: http://europepmc.org/abstract/MED/29986672; https://bmccancer.biomedcentral.com/track/pdf/; 10.1186/s12885-018-4660-7; https://europepmc.org/articles/PMC6038248; https://europepmc.org/articles/PMC6038248?pdf=render. 10.1186/s12885-018-4660-7 PMC6038248 29986672

[25] Lanke V, Trimm K, Habib B, Tamblyn R. Evaluating the effectiveness of mobile apps on medication adherence for chronic conditions: systematic review and meta-analysis. J Med Internet Res. 2025;27:e60822. 40743450 10.2196/60822 PMC12312993

[26] Liu J, Yu Y, Yan S, Zeng Y, Su S, He T, et al. Risk factors for self-reported medication adherence in community-dwelling older patients with multimorbidity and polypharmacy: a multicenter cross-sectional study. BMC Geriatr. 2023;23(1):75. 36740694 10.1186/s12877-023-03768-7 PMC9900971

[27] Ayele AA, Tegegn HG, Ayele TA, Ayalew MB. Medication regimen complexity and its impact on medication adherence and glycemic control among patients with type 2 diabetes mellitus in an Ethiopian general hospital. BMJ Open Diabetes Res Care. 2019;7(1):e000685. 31321061 10.1136/bmjdrc-2019-000685 PMC6606061

[28] Geissler J, Sharf G, Bombaci F, Daban M, De Jong J, Gavin T, et al. Factors influencing adherence in CML and ways to improvement: Results of a patient-driven survey of 2546 patients in 63 countries. J Cancer Res Clin Oncol. 2017;143(7):1167–76. 28289895 10.1007/s00432-017-2372-z PMC11819081

[29] Pluta A, Sulikowska B, Manitius J, Posieczek Z, Marzec A, Morisky DE. Acceptance of illness and compliance with therapeutic recommendations in patients with hypertension. Int J Environ Res Public Health. 2020;17(18). 10.3390/ijerph17186789 PMC7557862 32957678

[30] Marwa Mosaad A, Samah Elsayed G. Effectiveness of Health Education Program Regarding Foot Self-care on Risk for Developing Foot Ulcer Among Patients with Diabetes. Am J Nurs Sci. 2019;8(5):274–87.

[31] Gürgöze MT, van der Galiën OP, Limpens MAM, Roest S, Hoekstra RC, AS IJ, et al. Impact of sex differences in co-morbidities and medication adherence on outcome in 25 776 heart failure patients. ESC Heart Fail. 2021;8(1):63–73. 33247631 10.1002/ehf2.13113 PMC7835621

[32] Venditti V, Bleve E, Morano S, Filardi T. Gender-related factors in medication adherence for metabolic and cardiovascular health. Metabolites. 2023;13(10). 10.3390/metabo13101087 PMC10609002 37887412

[33] Zopf Y, Rabe C, Neubert A, Gassmann KG, Rascher W, Hahn EG, et al. Women encounter ADRs more often than do men. Eur J Clin Pharmacol. 2008;64(10):999–1004. 18604529 10.1007/s00228-008-0494-6

[34] Chen SL, Lee WL, Liang T, Liao IC. Factors associated with gender differences in medication adherence: a longitudinal study. J Adv Nurs. 2014;70(9):2031–40. 24506542 10.1111/jan.12361

[35] Wagoner ST, Schaefer MR, Rawlinson AR, Shapiro SK, Kavookjian J, Gray WN. Barriers to Treatment Adherence Among College Students with Attention-Deficit/Hyperactivity Disorder. J Dev Behav Pediatr. 2020;41(1):9–15. 31449195 10.1097/DBP.0000000000000723

[36] Li J, Steinberg J, Mynatt E, Mishra V. Navigating the paradox: challenges and strategies of university students managing mental health medication in real-world practices. arXiv [preprint]. 2024;abs/240807784. 10.2196/67579 PMC13002155 41855492

[37] Shammin Haque NNA, Mushroor S, Nusrat Sultana. Association between knowledge of self medication and adherence among medical students in Dhaka, Bangladesh. Res J Pharm Technol. 2017;10(5).

